# A Hydrogel Drink With High Fructose Content Generates Higher Exogenous Carbohydrate Oxidation and a Reduced Drop in Dental Biofilm pH Compared to Two Other, Commercially Available, Carbohydrate Sports Drinks

**DOI:** 10.3389/fnut.2020.00088

**Published:** 2020-06-12

**Authors:** Stefan Pettersson, Martin Ahnoff, Fredrik Edin, Peter Lingström, Charlotte Simark Mattsson, Ulrika Andersson-Hall

**Affiliations:** ^1^Department of Food and Nutrition, and Sport Science, Center for Health and Performance, University of Gothenburg, Gothenburg, Sweden; ^2^Maurten AB, Research and Development, Gothenburg, Sweden; ^3^Department of Cariology, Institute of Odontology, Sahlgrenska Academy, University of Gothenburg, Gothenburg, Sweden; ^4^Department of Physiology, Institute of Neuroscience and Physiology, Sahlgrenska Academy, University of Gothenburg, Gothenburg, Sweden

**Keywords:** dental caries, endurance athletes, sports nutrition, stable isotope, substrate oxidation

## Abstract

The purpose of this study was to evaluate the substrate oxidation of three commercially available, 14%-carbohydrate sports drinks with different compositions, osmolality, and pH for their impact on dental exposure to low pH. In a cross-over, randomized double-blinded design, 12 endurance athletes (age 31. 2 ± 7.7 years, $\dot{\text{V}}$O_2max_ 65.6 ± 5.0 mL·kg^−1^) completed 180 min of cycling at 55% W_max_. During the first 100 min of cycling, athletes consumed amylopectin starch (AP), maltodextrin+sucrose (MD+SUC), or maltodextrin+fructose hydrogel (MD+FRU) drinks providing 95 g carbohydrate·h^−1^, followed by water intake only at 120 and 160 min. Fuel use was determined using indirect calorimetry and stable-isotope techniques. Additionally, dental biofilm pH was measured using the microtouch method in a subsample of participants (*n* = 6) during resting conditions before, and at different time intervals up to 45 min following a single bolus of drink. Exogenous carbohydrate oxidation (CHO_EXO_) during the 2nd hour of exercise was significantly (*P* < 0.05) different between all three drinks: MD+FRU (1.17 ± 0.17 g·min^−1^), MD+SUC (1.01 ± 0.13 g·min^−1^), and AP (0.84 ± 0.11 g·min^−1^). At the end of exercise, CHO_EXO_ and blood glucose concentrations (3.54 ± 0.50, 4.07 ± 0.67, and 4.28 ± 0.47 mmol·L^−1^, respectively) were significantly lower post MD+FRU consumption than post MD+SUC and AP consumption (*P* < 0.05). Biofilm acidogenicity at rest demonstrated a less pronounced pH fall for MD+FRU compared to the acidulant-containing MD+SUC and AP (*P* < 0.05). In conclusion, while total intake of MD+FRU showed signs of completed uptake before end of monitoring, this was less so for MD+SUC, and not at all the case for AP. Thus, this study showed that despite carbohydrates being encapsulated in a hydrogel, a higher CHO_EXO_ was observed following MD+FRU drink ingestion compared to AP and MD+SUC consumption upon exposure to the acidic environment of the stomach. This finding may be related to the higher fructose content of the MD+FRU drink compared with the MD+SUC and AP drinks. Furthermore, a carbohydrate solution without added acidulants, which are commonly included in commercial sport drinks, may have less deleterious effects on oral health.

## Introduction

It is well-established that carbohydrate-electrolyte drink ingestion can enhance exercise performance and capacity (1, 2). Proposed ergogenic benefits include attenuation of dehydration, maintenance of euglycemia, and high carbohydrate oxidation rates. Thus, to enhance performance during competition and prolonged key training sessions, general fluid intake guidelines advocate frequent consumption of moderate volumes (~200 mL) of dilute (≤ 8%) sport drinks, while more concentrated (≥10%) carbohydrate solutions are primarily advocated for pre-competition, glycogen super-compensation or glycogen, and hydration status restoration post-exercise (3, 4).

However, considering that sweat rate and fluid requirements are dependent on exercise intensity, duration, and environmental conditions (e.g., altitude, heat, and humidity), intake of a more concentrated carbohydrate solution may also provide a practical strategy to sustain performance and high carbohydrate oxidation rates during exercise. On the other hand, increasing carbohydrate content of the ingested fluid has been shown to delay gastric emptying rate (5). Hypertonic drinks have also been suggested to cause water retention in the intestine and carbohydrate malabsorption which might increase the risk of gastrointestinal (GI) discomfort (6). While numerous studies have investigated substrate utilization and GI responses following ingestion of highly concentrated (>13%) carbohydrate drinks during exercise [as reviewed by Wilson (7)], these drinks were not commercially available and often ingested at rates well-above what is generally recommended during prolonged exercise (up to 90 g carbohydrates·h^−1^), thereby limiting applicability of the results.

The combined use of respiratory exchange and stable-isotope tracer techniques (mainly measuring ^13^C in exhaled CO_2_) is a common method to track and quantify exogenous carbohydrate fuel use during exercise (8). The main rate-limiting step in exogenous carbohydrate delivery to muscle during exercise is likely intestinal absorption, with strong evidence that the maximal rate of glucose and/or glucose polymer absorption is ~1 g·min^−1^ when ingested in excess of 1.2 g·min^−1^ (9). However, by ingesting mixtures of glucose and fructose, which are absorbed by different intestinal transporters [sodium-dependent glucose transporter 1 (SGLT1) and glucose transporter 5 (GLUT-5), respectively], exogenous carbohydrate oxidation rates can increase (10–12) up to ~1.7 g·min^−1^ (13). Studies with a sports perspective on exogenous carbohydrate oxidation generally have been designed with repeated carbohydrate intake and gas exchange measurements throughout the exercise period to estimate oxidation at steady-state conditions toward the end of the period (10, 11, 14–17). In contrast, early studies with a medical perspective on glucose uptake and oxidation used a single bolus administration of carbohydrate and monitoring over 4 h, which was found to suffice for oxidation of about 90% of ingested carbohydrate (18, 19). However, a study design including measurements of exogenous carbohydrate oxidation during repeated intake and beyond last intake of drinks with different carbohydrate compositions would not only provide information on how carbohydrates were utilized until the end of exercise, but also to which extent this differed between types of carbohydrate drink. To date, no such study has been performed.

Another aspect of carbohydrate-containing sport drinks has been highlighted by recent studies emphasizing the risk for elite athletes of developing poor oral health, including dental caries and erosion, which affects their quality of life and professional performance (20, 21). Although the etiology of both caries' incidence and risk for development of erosion is complex and multifactorial, frequent intake of fermentable carbohydrates and ingestion of drinks with low-pH and high buffering capacity are known to have a strong, negative influence. A recent pH assessment of sports drinks commercially available in the US found all products to be highly acidic (i.e., pH < 4) (22). This is in line with a previous investigation of sports products in Europe (23). That drinks with a pH below 4.0 have been described as potentially damaging to the dentition (24) raises concerns about the long- and short-term effects of repeated sports drink consumption (25).

The purpose of this cross-over, randomized, double-blinded study was to evaluate, and make comparisons between three highly concentrated (~14%) commercial sports drinks of different carbohydrate composition, osmolality, and pH, for substrate oxidation and their impact on GI symptoms and dental biofilm acidogenicity. At the time of designing the study (2017), only a limited number of highly concentrated sports drinks specifically marketed for use during exercise were commercially available. Based on previous research using a dual-isotope approach (15), we hypothesized that a novel, hydrogel-forming hypertonic solution containing maltodextrin-fructose (MD+FRU; 1:0.7 MD:FRU ratio), despite being encapsulated in a hydrogel upon exposure to the acidic environment of the stomach (26), would generate a higher rate of exogenous carbohydrate oxidation than an isotonic maltodextrin:sucrose (MD+SUC) drink (1:0.25 MD+SUC ratio) or single source hypotonic carbohydrate solution (amylopectin starch; AP) during the 2nd hour of exercise (60–120 min). By extending exercise until 80 min after the last carbohydrate intake (time = 100 min), this study was designed to gain additional understanding on differences in carbohydrate uptake and oxidation of the three investigated sports drinks. Furthermore, as the three drinks differed in acidity, we hypothesized that the MD+FRU drink containing no acidulants (pH 6.0) would, during resting conditions, result in a less pronounced fall in dental biofilm pH compared to the MD+SUC (pH 3.5) and AP (pH 3.9) drinks, both of which contained acidulants.

## Subjects and Methods

Twelve trained endurance cyclists/triathletes aged 31.2 ± 7.7 years (height: 183.3 ± 7.0 cm^−1^; body mass: 76.6 ± 3.3 kg^−1^; and $\dot{\text{V}}$O_2max_: 65.6 ± 5.0 mL·kg^−1^) were recruited in the Gothenburg region, Sweden via local contacts. One subject withdrew after completing two of the three experimental exercise trials for reasons unrelated to the study. A subsample (*n* = 6) of the participants was randomly selected for dental biofilm acidogenicity assessments performed under resting conditions. All participants provided written informed consent and the study was approved by the local ethics committee at Gothenburg University (Dnr. 1088-17).

### Experimental Design

**Figure 1:**
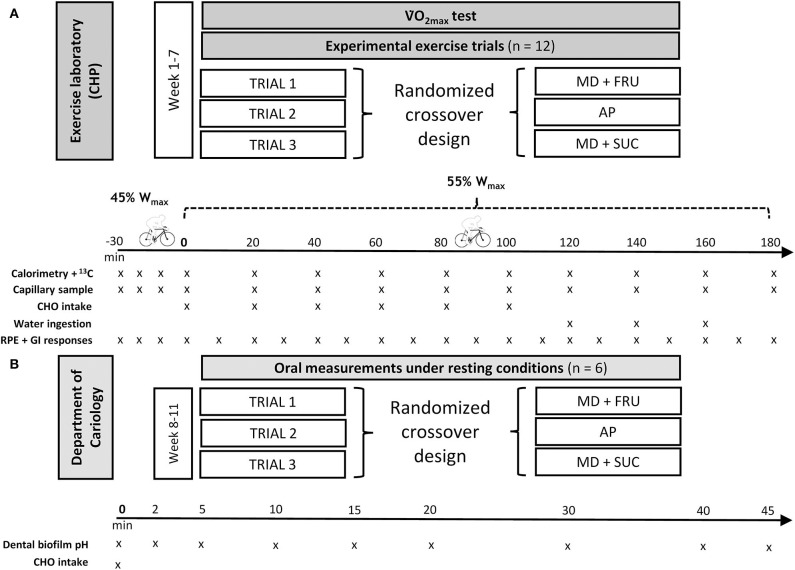
Flowchart study design and schematic of the **(A)** experimental exercise trials, and **(B)** oral measurements under resting conditions. MD+FRU, maltodextrin+fructose drink; MD+SUC, maltodextrin+sucrose drink; AP, amylopectin drink.

All study participants were required to attend four separate visits to the exercise laboratory [Center for Health and Performance (CHP), Gothenburg University] (see Figure 1A). At first visit, each participant performed an incremental cycle test to determine maximal oxygen uptake ($\dot{\text{V}}$O_2max_) and maximum power output (*W*_max_).

Within 2 weeks following the $\dot{\text{V}}$O_2max_ test, the participants performed the first of three exercise trials in randomized order. The following drinks were administered in a double-blind manner at each respective trial: AP (a 3:2 mixture of Vitargo Electrolyte® and Vitargo Pure® Swecarb AB, Sweden), MD+SUC (Isostar Endurance Plus® Wander-Isostar, Switzerland), or MD+FRU (Maurten 320® Maurten AB, Sweden). Vitargo Electrolyte was reinforced with pure amylopectin according to the manufacturer's recommendations in order to achieve an increased carbohydrate content without increasing the concentration of other drink components. Drink characteristics are shown in Table 1. Isotope analysis (FAU, Erlangen-Nürnberg, Germany) demonstrated a high natural abundance of ^13^C in the three drinks: AP = −11.71%0, MD+SUC = −14.67%0, and MD+FRU = −11.50%0 vs. Vienna Pee Dee Belemnite (VPDB), respectively.

**Table 1:** Drink characteristics.

	**MD+FRU**	**MD+SUC^†^**	**AP**
**Contents per serving (g)**			
Total carbohydrates	31.7	31.7	31.7
of which carbon	13.3	13.7	14.1
Relative carbon content	1.00	1.03	1.06
Maltodextrin	18.1	24.9	–
Starch	–	–	30.4
–Sucrose	–	6.8	–
Fructose	13.6	–	–
Sodium (Na^+^)	0.20	0.16	0.29
Water	201	224	201
Other ingredients	Alginate, pectin	Citric acid, Na-citrate, aroma, colorants, vitamins C, E, B1	Citric acid, Na-citrate, Ca-, K- and Mg-gluconate, aroma, colorant, acesulfam K, aspartame
pH	6.0	3.5	3.9
Osmolality^*^ (mOsm/kg H_2_O)	490	235	69

*MD+FRU, maltodextrin+fructose; MD+SUC, maltodextrin+sucrose; AP, amylopectin*.

* *Osmolality was measured using a Type 13 Autocal osmometer (Roebling Messentechnik, Bremen, Germany)*.

† *Sucrose and fructose content of the MD+FRU beverage was determined by HLPC-CAD (SYNLAB, Analytics & Services, Linköping, Sweden)*.

Each experimental trial involved the ingestion of 1.58 g·min^−1^ of carbohydrates (95 g·h^−1^) interspersed as six boluses provided every 20 min during a 100 min period with first feeding at time = 0 (i.e., 30 min after onset of exercise see under *Calculations* for rationale). The study design (Figure 1) included measurements during continuous exercise up to 80 min after the last intake of carbohydrates, allowing observations on the degree of uptake and oxidation completed during this time period to be made. Accordingly, during the last hour of exercise, participants received 200 ml of plain water on three occasions (time = 120, 140, and 160 min).

All exercise tests were performed on the same cycle ergometer (Monark 828e, Varberg, Sweden) and under the same environmental conditions (19.5 ± 0.6.C and 41.7 ± 14.1% relative humidity). Additionally, a subsample (*n* = 6) of participants made three separate visits in randomized order to the cariology lab for dental measurements during resting conditions following a bolus intake of one of the three sports drinks (Figure 1B).

### $\dot{\text{V}}$O_2max_ Test

The incremental exercise test consisted of a 5 min warm up and 3, 5 min, steady state work-loads, followed by a 22 W increase of load every minute until the participants reached volitional exhaustion or could not maintain pedal speed (≥90 revolutions·min^−1^). Maximal oxygen consumption ($\dot{\text{V}}$O_2max_) was considered to be reached when one of the following three criteria were met: (1) a leveling off of $\dot{\text{V}}$O_2_ with increasing workload; (2) a plateau of $\dot{\text{V}}$O_2_ with increasing workload; and (3) a respiratory exchange ratio (RER) of ≥1.08. $\dot{\text{V}}$O_2max_ calculated as the average oxygen uptake during the last 60 s of the test. Heart rate (Polar Electro OY, model S10i, Finland) and work load (W) were continuously recorded, and $\dot{\text{V}}$O_2_ and VCO_2_ were measured using an online automated gas analysis system (Jaeger Oxycon Pro Jaeger, Viasys Healthcare, Germany) calibrated according to manufacturer's instructions. The *W*_max_ (372 ± 33 W) was predicted from the $\dot{\text{V}}$O_2_ of each submaximal workload by extrapolation up to $\dot{\text{V}}$O_2max_ using linear regression.

### Diet and Activity Before Testing

In order to reduce background enrichment of expired VCO_2_, participants were instructed to perform a glycogen depletion session 7 days before the first experimental trial, and received verbal and written instructions to avoid ingestion of carbohydrates derived from maize/corn and sugar cane throughout the whole study period. Participants were asked not to perform vigorous exercise on the day before each experimental trial, to record their diet on the first of these days, and to repeat this diet on days before subsequent trials. Participants in the dental biofilm acidogenicity assessments were asked to refrain from tooth brushing and all other forms of oral hygiene during the last 24 h prior to each test session.

### Exercise Trials

The experimental exercise trials were performed in the morning in a fasted state (exercise start at 07:30). Prior to exercise, height, and nude body mass were recorded (Seca 764, Hamburg, Germany) and a resting blood sample was collected. Participants were subsequently mounted onto the cycle ergometer; the first 30 min of cycling was performed at 45% *W*_max_ (50.9 ± 2.0% $\dot{\text{V}}$O_2max_) and the remaining 180 min at 55% *W*_max_ (60.3 ± 2.5% $\dot{\text{V}}$O_2max_). Respiratory gases were collected for 5 min periods on 12 occasions: at every 10 min interval for the first 30 min of exercise, and then at 5 min intervals every 20 min interval (Figure 1). Mean values during the final 60 s at each measurement point were used to assess substrate utilization via RER.

For the measurement of ^13^C/^12^C in expired CO_2_, expired gas was withdrawn into two 65 mL syringes (Kendal Monoject, UK) connected to the mixing chamber (Jaeger, Germany) via a three-way valve. Samples were emptied from the syringe into 12 mL Exetainer vials (Labco Ltd, Lampeter, UK) in duplicate for later analysis.

Psychometric scales including overall rating of perceived exertion (RPE) (Borg category scale 6–20) and severity of GI symptoms (i.e., gas, bloating, abdominal discomfort, nausea, stomach rumbling, urgency to have a bowel movement, and abdominal pain), where symptoms were rated on a scale from 0 to 20 (no symptoms to worst conceivable symptoms) and level of digestive comfort (extremely uncomfortable to extremely comfortable) (27), were recorded at 10 min intervals throughout the 180 min 55% *W*_max_ exercise bout.

Fingertip blood sampling, drink ingestion, and psychometric scales (in that order) were performed immediately following the respiratory gas measurement.

### Oral Measurements Under Resting Conditions

The dental biofilm pH was assessed after a single exposure using the microtouch method, where a microelectrode (Beetrode, MEPH-1; WP Instruments, New Haven, Conn., USA) connected to an Orion SA720 pH/ISE Meter (Orion Research, Boston, Mass., USA) was inserted into the dental biofilm (28). Each drink (10 mL) was kept in the mouth for 1 min. Biofilm pH measurements were obtained from two tooth sites (upper left and right premolar-molar region). A salt bridge was established in a 3 M KCl solution between the reference electrode and one of the subject's fingers. Prior to and during each test session, the electrode was calibrated against a standard buffer at pH 7.00. The dental biofilm pH was measured before rinsing with 10 ml of the respective drink at baseline (0 min) and at different time points (2, 5, 10, 15, 20, 30, 40, and 45 min).

### Chemical Analysis

Blood samples were analyzed for glucose and lactate (Biosen C-line, EKF Diagnostics). Breath samples were analyzed for ^13^CO_2_/^12^CO_2_ enrichment (δ^13^C) using a Thermo Scientific Delta Ray isotope ratio infrared spectrometer (IRIS) with a Universal Reference Interface (URI) and a Teledyne CETAC ASX-7100 Autosampler. Every two samples were bracketed by calibrating gas (δ^13^C 27.8%0 VPDB). The ^13^C enrichment of drink contents was determined using a Costech Elemental Analyzer (ECS 4010; Costech International, Pioltello, Italy) in continuous flow mode coupled to a Thermo Scientific Delta V plus (ThermoFisher Scientific, Bremen, Germany) isotope ratio mass spectrometer (Friedrich-Alexander-Universität, Erlangen, Germany). All isotope ratio data were normalized to the VPDB scale.

### Calculation of Oxidation Rates and Exogenous Carbohydrate Oxidation Efficiency

Rates of total carbohydrate and fat oxidation (g·min^−1^) during the experimental trials were calculated from $\dot{\text{V}}$O_2_ and VCO_2_ (L·min^−1^) using stoichiometric equations (29) based on the assumption that protein oxidation during exercise was negligible.

(1) $$\begin{array}{r} \text{Carbohydrate }\left(\text{g}\cdot \text{mi}{\text{n}}^{-1}\right)=\left(4.585\times \overset{∙}{\text{V}}\text{C}{\text{O}}_{2}\right)-\left(3.226\times \overset{∙}{\text{V}}{\text{O}}_{2}\right) \end{array}$$

(2) $$\begin{array}{r} \text{Fat }\left(\text{g}\cdot \text{mi}{\text{n}}^{-1}\right)=\left(1.695\times \overset{∙}{\text{V}}\text{C}{\text{O}}_{2}\right)-\left(1.701\times \overset{∙}{\text{V}}{\text{O}}_{2}\right) \end{array}$$

The isotopic enrichment was expressed as %0 difference between δ^13^C/^12^C ratio of the sample and a known laboratory reference standard according to the formula of Craig (30):

(3) $$\begin{array}{r} {\text{δ}}^{13}\text{C}=\left(\left(\frac{13\text{C}/12\text{C sample}}{13\text{C}/12\text{C standard}}\right)-1\right)\;\cdot 10^{3} \end{array}$$

The δ^13^C was then related to an international standard (VPDB). The rate of exogenous carbohydrate oxidation was calculated using the formula of Mosora et al. (31):

(4) $$\begin{array}{r} \text{Exogenous carbohydrate oxidation }\left(\text{g}\cdot \text{mi}{\text{n}}^{-1}\right)\\ ={\text{VCO}}_{2}\;\times \;\left(\frac{\text{δExp}-\text{δEx}{\text{p}}_{\text{ref}}}{\text{δIng}-\text{ δEx}{\text{p}}_{\text{ref}}}\;\right)\left(\frac{1}{\text{k}}\right) \end{array}$$

where δExp is the isotopic composition of expired CO_2_ during exercise, δIng is the isotopic composition of ingested drink, δExp_ref_ is isotopic composition of expired CO_2_ 30 min after the onset of exercise, and *k* (0.7467) is the amount of CO_2_ (L^−1^) produced for the complete oxidation of one gram of glucose.

For the purpose of serving as a warmup, but foremost to reach a physiological steady state condition and to obtain a representative baseline value for the ^13^C/^12^C ratio in breath air, the participants exercised for the first 30 min at 45% *W*_max_ in a fasted state (32). Data from a separate experiment (Supplementary Table 1), where the participants (*n* = 12) performed a 60 min exercise trial in a fasted state (30 min at 45% *W*_max_ followed by 30 min exercise at 55% *W*_max_), showed no mean difference in δ^13^C despite differences in work load between time = 30 and 60 min, respectively. Time = 0 min denotes the first carbohydrate feeding (Figure 1). Endogenous carbohydrate oxidation was calculated by subtracting exogenous carbohydrate oxidation from total carbohydrate oxidation.

The carbon content of a serving (Table 1) was calculated from the carbohydrate composition of each drink. Carbon content of fructose (FRU), sucrose (SUC), maltodextrin (MD), and amylopectin (AP) was calculated using the general chemical formula C_6n_H_10n+2_O_5n+1_: FRU (*n* = 1) 0.40 g/g; SUC (*n* = 2) 0.421 g/g; MD (*n* = 5.5 corresponding to dextrose equivalent DE = 18) 0.436 g/g; and AP (*n* = 4,000–6,000) 0.444 g/g, respectively.

Oxidation efficiency was calculated as the ratio between the total exogenous carbohydrate oxidation during the trial (AUC_CHO_EXO_) and the total intake of carbohydrates, both expressed as the corresponding amount of carbon.

(5) $$\begin{array}{r} \text{ MD}+\text{FRU carbon content}\\ =0.420\times \text{weight of carbohydrates} \end{array}$$

(6) $$\begin{array}{r} \text{ MD}+\text{SUC carbon content}\\ =0.432\times \text{weight of carbohydrates} \end{array}$$

(7) $$\begin{array}{r} \text{ AP carbon content}\\ =0.444\times \text{weight of carbohydrates} \end{array}$$

### Statistical Analyses

#### Power Calculation

Sample size (*n*) was estimated via G^*^ software (Version 3.1.9.2, Universität Düsseldorf, Germany). Assuming a power of 90% and an α-level of 5%, *n* = 9 participants was determined to be adequate to detect differences in mean 0–120 min exogenous carbohydrate oxidation between trials. However, since this estimation was predicted from a limited sample size pilot study (*n* = 3; unpublished data), we decided to include 12 participants in this study. Furthermore, based on previous research by Hans et al. (33) demonstrating differences in salivary pH 5 min after a single bolus intake of sugary drinks with an intrinsic pH range (pH = 3.6–6.0) similar to that of the sport drinks included in the present study, *n* = 6 participants was determined to be adequate to detect differences between drinks.

### General Statistics

All data were checked for normality using a Shapiro–Wilk test. Two-way repeated measures ANOVA (treatment × time) with Bonferroni *post-hoc* adjustment was used to identify the location of significant differences (*P* < 0.05) when the analysis of variance yielded a significant *F*-ratio. All data were analyzed using SPSS 22.0 software (IBM, New York, USA). All data, unless otherwise stated, are presented as mean ± SD. Reproducibility of isotope enrichment measurements was assessed by comparing δ^13^C values (*X*1 and *X*2) from duplicate series of samples analyzed on different occasions, each series containing thirteen samples. For each series of duplicate samples, standard deviation of first measurement minus second measurements (σ_X1−*X*2_) was calculated. Reproducibility of single measurements was estimated as σ = σ_X1−*X*2_ × √2^−1^.

## Results

### $\dot{\text{V}}$O_2_, RER, Total Carbohydrate, and Fat Oxidation

Data on fat and carbohydrate oxidation are shown in Table 2. No significant differences were observed in energy expenditure (data not shown), RER, total fat oxidation (FAT), and total carbohydrate oxidation (CHO_TOT_) between the three experimental trials. A higher mean oxygen uptake was observed in the MD+FRU trial compared to that of the MD+SUC during the 1st hour of exercise and between AP and MD+SUC at 60–120 min and 0–180 min. These differences were significant (*P* < 0.05), but small (mean differences ≤ 0.07 L·min^−1^; see Table 2).

**Table 2:** Mean ± SD oxygen uptake ($\dot{\text{V}}$O_2_), respiratory exchange ratio (RER), and substrate oxidation during the 180 min cycling exercise at 55% *W*_max_ with ingestion of the three sports drinks.

		**MD+FRU (*n* = 12)**	**MD+SUC (*n* = 12)**	**AP (*n* = 11)**	***P*-value**		
	**Time (min)**	**Mean ± SD**	**Mean ± SD**	**Mean ± SD**	**MD+FRU vs. MD+SUC**	**MD+FRU vs. AP**	**MD+SUC vs. AP**
$\dot{\text{V}}$O_2_ (L·min^−1^)	20–60	3.04 ± 0.22	2.98 ± 0.23	3.04 ± 0.23	**0.008**	0.713	0.053
	60–120	3.05 ± 0.23	2.99 ± 0.26	3.06 ± 0.23	0.143	0.553	0.034*
	120–180	3.05 ± 0.23	3.02 ± 0.25	3.11 ± 0.28	0.463	0.183	0.060
	20–180	3.05 ± 0.22	3.00 ± 0.25	3.07 ± 0.24	0.122	0.328	**0.014**
RER	20–60	0.83 ± 0.03	0.84 ± 0.04	0.82 ± 0.01	0.397	0.838	0.339
	60–120	0.84 ± 0.02	0.84 ± 0.03	0.83 ± 0.01	0.931	0.171	0.246
	120–180	0.84 ± 0.02	0.85 ± 0.03	0.83 ± 0.02	0.812	0.371	0.209
	20–180	0.84 ± 0.02	0.84 ± 0.03	0.83 ± 0.01	0.682	0.350	0.207
CHO_TOT_ (g·min^−1^)	20–60	1.77 ± 0.42	1.82 ± 0.51	1.65 ± 0.18	0.619	0.799	0.472
	60–120	1.94 ± 0.31	1.91 ± 0.41	1.75 ± 0.19	0.686	0.175	0.348
	120–180	1.92 ± 0.23	1.97 ± 0.35	1.83 ± 0.29	0.893	0.614	0.491
	20–180	1.88 ± 0.28	1.90 ± 0.40	1.75 ± 0.20	0.894	0.427	0.362
FAT (g·min^−1^)	20–60	0.86 ± 0.18	0.81 ± 0.20	0.90 ± 0.09	0.191	0.892	0.217
	60–120	0.80 ± 0.14	0.78 ± 0.18	0.87 ± 0.11	0.844	0.156	0.117
	120–180	0.81 ± 0.14	0.77 ± 0.16	0.87 ± 0.11	0.704	0.263	0.085
	20–180	0.82 ± 0.14	0.79 ± 0.17	0.88 ± 0.11	0.495	0.297	0.098
CHO_ENDO_ (g·min^−1^)	20–60	1.16 ± 0.40	1.32 ± 0.54	1.22 ± 0.23	0.174	0.115	0.960
	60–120	0.77 ± 0.27	0.90 ± 0.41	0.91 ± 0.21	0.134	**0.002**	0.270
	120–180	0.84 ± 0.26	0.83 ± 0.35	0.81 ± 0.18	0.656	0.800	0.395
	20–180	0.93 ± 0.29	1.00 ± 0.41	0.97 ± 0.18	0.506	0.121	0.492
CHO_EXO_ (g·min^−1^)	20–60	0.60 ± 0.12	0.50 ± 0.12	0.43 ± 0.09	0.071	**0.001**	**0.044**
	60–120	1.17 ± 0.17	1.01 ± 0.13*	0.84 ± 0.11	**0.015**	**<0.001**	**<0.001**
	120–180	1.08 ± 0.09	1.14 ± 0.08	0.98 ± 0.12	0.218	**0.037**	**<0.001**
	20–180	0.95 ± 0.08	0.90 ± 0.08	0.77 ± 0.09	0.300	**0.001**	**<0.001**
AUC CHO_EXO_ (g)	0–60	26 ± 6	22 ± 5	19 ± 4	0.117	**0.003**	0.061
	60–120	71 ± 11	61 ± 7	51 ± 6	**0.018**	**0.001**	**<0.001**
	120–180	66 ± 6	69 ± 5	59 ± 7	0.411	**0.020**	**<0.001**
	0–180	163 ± 16	152 ± 16	129 ± 15	0.15	**<0.001**	**<0.001**
Maximal CHO_EXO_ (g·min^−1^)		1.34 ± 0.16	1.23 ± 0.12	1.03 ± 0.11	0.053	**<0.001**	**<0.001**
*T*_max_ (min)		125 ± 16	164 ± 15	165 ± 20	**<0.001**	**0.001**	0.796

*CHO_TOT_, total carbohydrate oxidation; FAT, fat oxidation; CHO_ENDO_, endogenous carbohydrate oxidation; CHO_EXO_, exogenous carbohydrate oxidation; T_max_, Time for Maximal CHO_EXO_; AUC, Area under the curve; MD+FRU, maltodextrin+fructose drink; MD+SUC, maltodextrin+sucrose drink; AP, amylopectin drink. P-values < 0.05 are displayed in bold*.

### Exogenous and Endogenous Carbohydrate Oxidation

**Figure 2:**
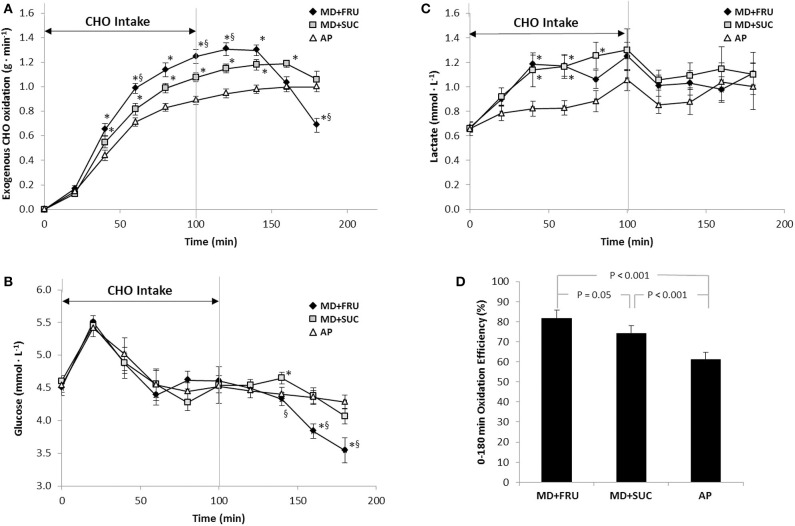
Mean ± SEM **(A)** exogenous carbohydrate oxidation rates (g·min^−1^), **(B)** blood glucose concentration (mmol·L^−1^), **(C)** blood lactate concentrations (mmol·L^−1^) during and after repeated intake of the three sports drinks, and **(D)** exogenous carbohydrate oxidation efficiencies during the 180 min exercise (*n* = 12). *Significant difference vs. AP, ^§^significant difference vs. MD+SUC. Exogenous carbohydrate oxidation efficiencies **(D)** were calculated by dividing total exogenous carbohydrate oxidation (gram carbon) with total carbohydrate intake (gram carbon) of respective sports drink formulation. MD+FRU, maltodextrin+fructose drink; MD+SUC, maltodextrin+sucrose drink; AP, amylopectin drink.

Exogenous carbohydrate oxidation (CHO_EXO_) for each participant (Supplementary Table 2) was calculated from changes in δ^13^CO_2_ of breath samples taken during the 3.5 h cycling exercise (Supplementary Figure 1). Reproducibility of δ^13^CO_2_ measurements was at an average 0.06‰ (range 0.03–0.09‰). Mean values for CHO_EXO_ oxidation are presented in Table 2 (±SD) and Figures 2A,D (±SEM). During the first 2 h of exercise (0–120 min), there was a gradual and significant increase in CHO_EXO_ across trials [Figure 2A, main effect of time; *F*_(6, 60)_ = 484.8; *P* < 0.001]. The CHO_EXO_ was significantly higher for MD+FRU compared with both MD+SUC and AP [Figure 2A; interaction effect; *F*_(12, 120)_ = 10.3; *P* < 0.001]. The CHO_EXO_ for MD+SUC was also significantly higher than for AP.

When examining the 2nd hour of exercise (Table 2), mean CHO_EXO_ oxidation was 16 and 39% higher following MD+FRU ingestion compared to MD+SUC and AP trials, respectively. The AP drink generated significantly lower peak CHO_EXO_ oxidation (defined as the mean of the highest value reached by each individual) compared to MD+SUC and MD+FRU, and there was a tendency (*P* = 0.053) toward MD+FRU generating higher CHO_EXO_ oxidation than MD+SUC. In addition, peak CHO_EXO_ oxidation occurred ~40 min earlier for MD+FRU than MD+SUC and AP (*P* < 0.0001; Table 2). However, in many cases for MD+SUC, and in all cases for AP, a *T*_max_ in the CHO_EXO_ vs. time curve could not be distinguished within the 180 min exercise period.

After replacing the carbohydrate feedings with water (i.e., from 120 min onwards), a significant time × treatment interaction was also evident [*F*_(6, 60)_ = 15.4; *P* < 0.001] and mean CHO_EXO_ oxidation was significantly lower in MD+FRU trial than MD+SUC and AP at the end of exercise (*P* = 0.001 and 0.003, respectively). As shown in Figure 2A, CHO_EXO_ oxidation did not level-off in the AP trial despite the withdrawal of carbohydrate feedings, nor was it attenuated at 140 and 160 min in the MD+SUC trial. However, the total amount of the ingested carbohydrates that was oxidized during the entire 180 min exercise was significantly less following AP than MD+FRU and MD+SUC ingestion [−34 g, 95% confidence interval (CI) = −49 to −20 g, *P* < 0.001, and −24 g, 95% CI: −31 to −17 g, *P* < 0.001, respectively] (Figure 2D). In summary, exogenous oxidation rates for AP, and to a smaller extent MD+SUC, were lower than the oxidation rate for MD+FRU during the first 140 min, but higher than MD+FRU toward the end of the trial.

Endogenous carbohydrate oxidation (CHO_ENDO_) during exercise was similar across trials (Table 2), except during the 2nd hour of exercise when significantly more CHO_ENDO_ was utilized in the AP trial compared to the MD+FRU trial (mean difference 0.19 g·min^−1^; 95% CI: 0.08–0.30 g, *P* = 0.003). This may have been related to the significantly lower CHO_EXO_ oxidation in the AP trial during this time period (mean difference 0.34 g·min^−1^; 95% CL 0.19–0.49 g, *P* = 0.001).

### Blood Glucose and Lactate Concentrations

Blood glucose concentrations were similar between MD+FRU, MD+SUC, and AP trials before and during exercise when subjects were fed carbohydrates (Figure 2C). During the last hour of exercise (e.g., min 120–180, water ingestion only), a trial × time interaction between drink conditions was detected [*F*_(6, 54)_ = 7.40; *P* = 0.003]. Between 140 and 180 min of exercise, glucose concentrations decreased more in the MD+FRU trial than in the MD+SUC and AP trials, reaching statistical significance at 140, 160, and 180 min for MD+SUC (*P* = 0.015, 0.004, and 0.04, respectively) and for AP at 160 and 180 min (*P* = 0.004 and *P* < 0.001, respectively). Glucose concentrations at the end of exercise were 3.54 ± 0.50, 4.07 ± 0.67, and 4.28 ± 0.47 mmol·L^−1^ for MD+FRU, MD+SUC, and AP, respectively.

Blood lactate concentrations did not differ between drink conditions at the onset of the 55% *W*_max_ exercise bout (0.67 ± 0.14, 0.66 ± 0.19, and 0.66 ± 0.18 mmol·L^−1^ for MD+FRU, MD+SUC, and AP, respectively) and reached peak values across trials synchronously with the last carbohydrate feeding at 100 min (Figure 2B). Between 20 and 100 min, lactate concentrations were consistently lower in the AP trial compared to MD+FRU and MD+SUC trials, reaching statistical significance at 40, 60 min for MD+FRU (*P* = 0.001, 0.002, respectively) and for MD+SUC at 40, 60, and 80 min (*P* = 0.032, 0.028, and 0.015, respectively).

### Gastrointestinal Symptoms, Heart Rate, RPE, and Body Mass Change

**Figure 3:**
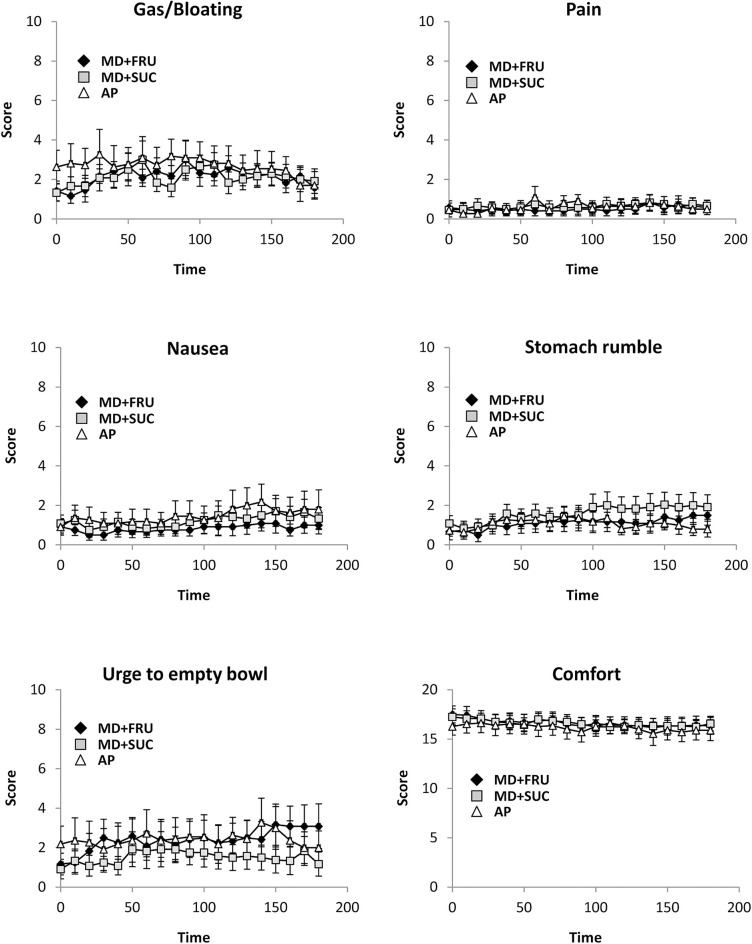
Mean ± SEM perceptions of gastrointestinal (GI) symptoms during the 180 min exercise (*n* = 12). For all GI symptoms except GI comfort: 0, no symptoms; 20, worst conceivable symptoms; for GI comfort: 0, extremely uncomfortable; 20, extremely comfortable (10 = neutral). MD+FRU, maltodextrin+fructose drink; MD+SUC, maltodextrin+sucrose drink; AP, amylopectin drink.

**Figure 4:**
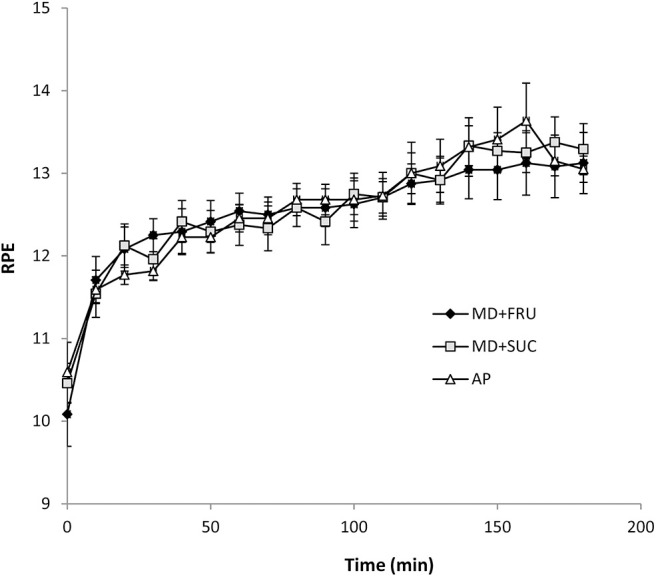
Mean ± SEM ratings of perceived exertion (RPE) during the 180 min exercise (*n* = 12). MD+FRU, maltodextrin+fructose drink; MD+SUC, maltodextrin+sucrose drink; AP, amylopectin drink.

All six measures of GI symptoms were at the low end of the 20-point scale (where 20 = worst conceivable symptoms) with mean values of respective measures < 4, and no observed differences across the three trials (Figure 3). Subjective RPE gradually increased over time [*F*_(18, 180)_ = 24.9; *P* < 0.001] with no difference between drink conditions. Mean RPE in the final hour of exercise was 13.1 ± 0.1, 13.2 ± 0.2, and 13.3 ± 0.2 for MD+FRU, MD+SUC, and AP, respectively (Figure 4). The relative change in body mass (as percent of total body mass) was not significantly different across trials (1.1 ± 0.7, 0.7 ± 0.8, and 1.1 ± 0.5% for MD+FRU, MD+SUC, and AP, respectively).

### Oral Measurements Under Resting Conditions

**Figure 5:**
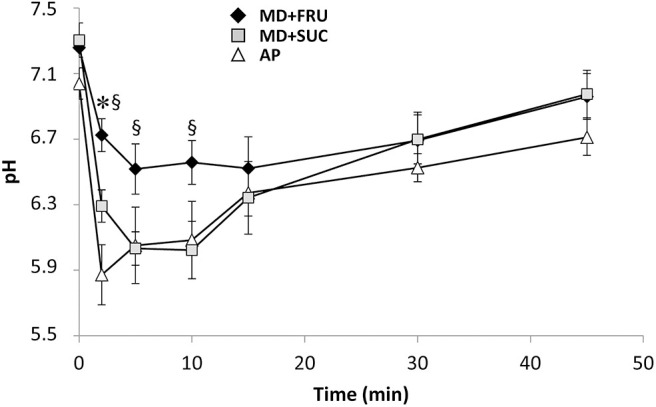
Mean ± SEM pH of dental biofilm during resting conditions measured before and 2–45 min after a single intake of carbohydrate drink (*n* = 6). *Significant difference vs. AP; ^§^significant difference vs. MD+SUC. MD+FRU, maltodextrin+fructose drink; MD+SUC, maltodextrin+sucrose drink; AP, amylopectin drink.

At 2–10 min after ingestion, dental biofilm pH was lowered to approximately pH 6.0 for the MD+SUC and AP drinks, while a less pronounced fall in pH was seen for MD+FRU (Figure 5). Statistically significant differences were found between MD+FRU and MD+SUC at 2 min [*F*_(2, 10)_ = 58.5; *P* = 0.001], 5 min [*F*_(2, 10)_ = 8.45; *P* = 0.034] and 10 min [*F*_(2, 10)_ = 17.8; *P* = 0.014], and between MD+FRU and AP at 2 min [*F*_(2, 10)_ = 58.5; *P* = 0.029]. The pH gradually recovered over a 10–15 min period, and from 15 min onwards no significant differences were seen between the three drinks. No statistically significant differences were found when comparing the AP and MD+SUC drinks. A larger maximum pH-drop [*F*_(2, 10)_ = 11.8; *P* = 0.004] and a lower minimum pH [*F*_(2, 10)_ = 51.4; *P* < 0.001] was found for MD+SUC compared to MD+FRU.

## Discussion

The present study investigated the effects of three highly concentrated, commercially available sports drinks differing in carbohydrate composition, osmolality, and acidity (pH) on substrate utilization and dental biofilm pH. As hypothesized, during the 2nd hour of carbohydrate feeding (60–120 min), the MD+FRU drink generated significantly higher exogenous carbohydrate oxidation than both the multiple transportable MD+SUC drink, and the single carbohydrate source drink (AP). Furthermore, biofilm-pH measurements at resting conditions demonstrated a significantly less pronounced fall for MD+FRU compared to that of both MD+SUC and AP.

### Exogenous Carbohydrate Oxidation

Previous research has indicated that there is a dose-response relationship between carbohydrate ingestion rate, exogenous carbohydrate oxidation, and performance during prolonged exercise (17, 34, 35). Thus, although performance was not measured in the present study, information on exogenous oxidation of commercially available sports drinks with different carbohydrate contents ingested at the recommended rate during prolonged exercise should be of relevance for athletes and sports nutrition practitioners.

The ingestion of MD+FRU resulted in a significantly (30%) higher peak CHO_EXO_ oxidation rate than AP and a tendency (*P* = 0.053) toward a 9% higher peak CHO_EXO_ oxidation rate compared to MD+SUC. The peak CHO_EXO_ rate observed following AP ingestion is in line with the well-accepted maximal absorption rate (~1 g·min^−1^) of ample glucose or glucose polymer solutions when fed in excess of 1.2 g·min^−1^ (16, 36). While this limitation in exogenous oxidation mainly is attributed to the saturation of intestinal SGLT1 transporters, co-ingestion of fructose, provided either as a monosaccharide or as a constituent of sucrose, is known to further increase oxidation rates by independent absorption by GLUT5 transporters in the intestine (37, 38). Consequently, the CHO_EXO_ oxidation rate was significantly higher in the MD+SUC trial than AP at all time points between 40 and 160 min of exercise, and reached a similar peak CHO_EXO_ oxidation rate as reported by Jentjens et al. (14) (1.25 ± 0.07 g·min^−1^), where subjects received a 2:1 glucose-sucrose mixture providing a total of 1.8 g of carbohydrates·min^−1^.

The peak CHO_EXO_ oxidation rate in the MD+FRU trial (1.34 ± 0.16 g·min^−1^) is comparable to oxidation rates achieved in other studies with similar intake levels and ratio of multiple transportable carbohydrate solutions (10, 11). Using a dual-isotope (^14^C-fructose and ^13^C-maltodextrin) approach, O'Brien et al. (15) demonstrated that a glucose/maltodextrin:fructose ratio of 1:0.8 providing 1.5 g carbohydrates·min^−1^ generated a higher total CHO_exo_ oxidation than an isocaloric 1.0:0.5 glucose/maltodextrin:fructose solution, a difference primarily attributed to the higher fructose oxidation efficiency of the 1.0:0.8 glucose/maltodextrin:fructose solution. In line with the findings of O'Brien et al. (15), a recent review suggested that the most worthwhile gains in exogenous oxidation and absorption are most likely to occur with a 1.0:0.7 glucose/maltodextrin:fructose solution (39). Thus, the tendency (*P* = 0.053) toward higher peak CHO_EXO_ oxidation rate for MD+FRU than for MD+SUC in the present study could be explained by the MD:SUC solution (1:0.25) providing a smaller amount of fructose (~0.17 g·min^−1^) for intestinal absorption and subsequent oxidation. For MD+FRU intake, it might be hypothesized that formation of a hydrogel in the stomach could affect gastric emptying rate and/or attenuate intestinal carbohydrate absorption.

Attenuating effects have been shown when supplementing meals with 5 g of sodium alginate with the aim of increasing satiety and decreasing postprandial glucose response in subjects with type-2 diabetes (40). The amount of alginate used, 5 g per meal, was substantially higher than the amount of alginate and pectin in this study (about 0.4 g per serving). On the other hand, it has been shown that adding pectin (1.4 g) to an enteral solution (total volume 490 mL), thereby increasing its viscosity, resulted in an increased gastric emptying rate in healthy individuals (41). This finding is in accord with a recent study where ingestion of a 500 mL bolus of a 18% carbohydrate drink containing 0.4 g pectin and 0.6 g of alginate resulted in 1.8 and 2.4 times faster gastric half-emptying time compared to an isoenergetic MD+FRU drink and a glucose+FRU drink, respectively (42). Recent studies have found no difference in total substrate oxidation and blood glucose concentrations (43–45) or exogenous carbohydrate oxidation (46) with the addition of sodium alginate and pectin in drinks matched for maltodextrin and fructose content during exercise.

It is well-established that compared with placebo or water intake, exogenous carbohydrate provision during prolonged exercise increases total carbohydrate oxidation, decreases fat oxidation and reduces the oxidation of endogenous carbohydrate, and that these alterations in substrate metabolism are entirely attributed to the oxidation of ingested (i.e., exogenous) carbohydrates (10, 13, 14). Less is known with regard to carbohydrate metabolism following cessation of intake of different carbohydrate sources during exercise. The difference between the present study design and a conventional design should be considered when interpreting the exogenous carbohydrate oxidation data obtained here. With a conventional study design, carbohydrates are supplied throughout the entire trial, and oxidation efficiencies are estimated from oxidation rates observed toward trial end, assuming steady state conditions for carbohydrate intake, uptake and oxidation output (9). The design used in this study encompasses exercise for 180 min from the start of carbohydrate intake, with carbohydrate intake at 20 min intervals during the first 100 min followed by intake of water only. With this design, the first 120 min period can be used for conventional carbohydrate oxidation evaluation, while the additional exercise period up to 180 min provides information on how carbohydrates, ingested during the first 100 min, were utilized up to 180 min, and to which extent this differed between types of drink (and between individuals).

For the majority of the 12 participants exercising with intake of MD+FRU, a distinct decrease in exogenous carbohydrate oxidation rate was observed after 120 or 140 min (Supplementary Table 2), presumably due to the high oxidation efficiency of ingested carbohydrates. For trials with MD+SUC, a decrease in oxidation rate was also evident after cessation of carbohydrate intake, but this occurred later than that which followed MD+FRU intake. For trials with AP, no decrease in oxidation rate was observed during the 180 min exercise period, indicating that intestinal uptake was not yet declining.

The relative total amount of the ingested carbohydrate that was oxidized during the 180 min exercise was significantly less for AP compared to MD+SUC and MD+FRU (61, 74, and 82%, respectively). Not only exogenous carbohydrate oxidation, but also lactate concentrations were consistently higher following ingestion of the fructose-containing drinks (MD+FRU and MD+SUC) than in the AP trial until cessation of carbohydrate feedings (100 min). This finding is most likely explained by the fact that fructose, after intestinal uptake, undergoes a two-step metabolic conversion, resulting in release of glucose and lactate into the circulation, both of which will be taken up and utilized as fuel in the working muscles (47). Thus, not only the dual mechanism for intestinal uptake of multiple transportable carbohydrates, but also the uptake into the muscles of lactate in addition to glucose, lies behind the higher maximal exogenous carbohydrate oxidation observed for MD+FRU and MD+SUC compared to AP.

The difference in oxidation efficiency between trials was also reflected in the blood glucose concentrations during the 100–180 min fasted state exercise, as glucose in the AP trial only declined marginally toward the end of this period compared to MD+SUC, and especially MD+FRU. The maintenance of glucose levels throughout the AP trial, despite no carbohydrate consumption during the latter part of the exercise (100–180 min), is indicative of residual carbohydrates in the GI tract progressively entering the circulation. In line with this assumption, McConell et al. (48) demonstrated that when subjects were fed a total of 200 g glucose during 120 min of moderately intense exercise, only ~70 g appeared as gut-derived glucose (i.e., entrance of carbohydrates from the gut into the systemic circulation). Likewise, rates of glucose appearance has been shown not to exceed ~1 g·min^−1^ and even to completely block hepatic glucose output at very large glucose intakes (3 g·min^−1^) (49). Furthermore, work by Pallikarakis et al. (50) also indicates a relationship between exogenous carbohydrate oxidation rate and subsequent decrements in blood glucose concentrations following cessation of carbohydrate intake. In that study, participants exercised for 270 min while ingesting either 25 g or 50 g doses of glucose (total ingestion rate 0.7 g and 1.5 g·min^−1^, respectively) every 30 min up to 210 min. During the final 30 min of exercise a more pronounced fall in blood glucose concentrations was evident with the lower glucose intake rate (not reaching the upper limits of intestinal SLGT1 saturation capacity), compared to the high glucose trial, indicating that some glucose remained in the GI tract in the latter condition. Significant differences between trials in plasma FFA, glycerol, and insulin concentrations were also noted. In the present study, oxidation efficiency and exogenous carbohydrate oxidation data suggest that MD+FRU is rapidly oxidized, proposing that after cessation of carbohydrate intake a gradual switch from exogenous to endogenous carbohydrate oxidation is initiated. Indeed, endogenous carbohydrate oxidation increased from 120 to 180 min in the MD+FRU trial (*P* = 0.002 between time points, data not shown), a development not seen for the other two trials. The drop in plasma glucose toward the end of the MD+FRU trial indicates that hepatic glucose output was not sufficient to maintain plasma glucose homeostasis during increased reliance on endogenous carbohydrate, probably due to insufficient hepatic glycogenolysis (51). In future studies of similar design, it would be of high interest to measure factors that regulate hepatic glycogenolysis and gluconeogenesis, i.e., insulin, glucagon, catecholamines and metabolites.

Although low oxidation efficiency/residual carbohydrates in the GI tract are suggestive of increasing the risk for GI symptoms during prolonged exercise (9), no adverse symptoms across the trials were reported. The absence of GI distress may be attributable to the exercise modality, exercise intensity, temperate environmental conditions (≤ 21.C) chosen in this study, and the fact that carbohydrate supplementation was limited to six intakes (190 g) during the 100 min period.

### Measurements of Oral Health

Research over the last decades has shown that the damage to the mineralized structures of the teeth is, for both dental caries and erosion, caused by pH-dependent mechanisms (52). A single intake of the three drinks showed that MD+FRU induced a less pronounced, maximal pH fall and a faster pH recovery compared to the other drinks. This suggests a reduced risk for enamel and dentin demineralization and thus reduced potential cariogenicity. In contrast, MD+SUC and AP reduced the pH below the critical pH (6.2) for demineralization of dentinal surfaces (53) during the first 10 min of the trial. A gradual recovery toward resting pH was noted for all three products during 10–45 min after the single carbohydrate challenge, which corroborates previous research showing that acid clearance and intraoral pH stabilization occur rapidly over 2–13 min (33, 54, 55). The difference in pH drop can probably be contributed to the added acidulants and low pH of MD+SUC and AP drinks. The larger drop in pH may, with frequent consumption, also increase the risk for erosive tooth wear (25). It is clearly shown that both the variation in content regarding carbohydrates and acidulantes has an impact on the pH pattern following ingestion. The pattern of the pH-curve of MD+FRU indicates that this drink, with fructose as carbohydrate source and no acidulantes, has both lower cariogenic and erosive potential compared with the other two products. Although the mean differences obtained may seem small, they can play an important role for dental health, particularly during frequent consumption and dry mouth conditions which are both relevant to individuals during intense exercise.

### Limitations

In this study, 6 × 31.7 g of carbohydrates (MD+FRU, MD+SUC, or AP) was served during the trials. Since monosaccharides (fructose, glucose), disaccharides (sucrose), and polysaccharide (amylopectin) differ in carbon and thus energy content, the carbon content per serving of the three drinks varied from 13.3 g (MD+FRU) to 13.7 g (MD+SUC) and 14.1 g (AP) (Table 1). Whilst it would have been possible to adjust the volume of each drink to achieve iso-energetic servings, this was not done. Therefore, compared to MD+FRU, the carbon content of MD+SUC and AP was greater (by 3 and 6%, respectively). However, for calculation of oxidation efficiency, the carbon content of ingested carbohydrates was accounted for. Calculating oxidation efficiencies without taking carbon content into account would have rendered too high values by a factor 1.05 (MD+FRU), 1.08 (MD+SUC), and 1.11 (AP), respectively. It is not evident to what extent differences in carbon content have been considered in other reported studies on exogenous carbohydrate oxidation evaluating different types of mono-di- and/or poly-saccharides. Furthermore, exogenous carbohydrate oxidation for ingested MD+SUC may have been underestimated if the oxidation efficiency of ingested sucrose was markedly higher than for ingested maltodextrin. The resulting error was estimated to be in the order of 1% (see Supplementary Text).

Peronnet et al. (56) have highlighted a weakness in current methods used to determine exogenous carbohydrate oxidation. They have stated that the proposed and commonly used equation does not take into account that exercise and/or exogenous substrate ingestion modify the composition of the mixture of endogenous substrates oxidized and, consequently, the isotopic composition of CO_2_ arising from oxidation of endogenous substrates. Thus, calculation methods using reference δ^13^C data from a separate trial without carbohydrate ingestion for each time point, or using δ^13^C measured in the same trial prior to start of exercise (i.e., at one single time point) as a reference (δExp_*ref*_ in Equation 4) both suffer from the limitation that endogenous substrates are not utilized in the same proportions when δExp and δExp_*ref*_ are measured. While absolute values of calculated oxidation rates may be affected by choice of the reference values δExp_*ref*_ (Equation 4), mean differences between trials will not be affected, as long as the reference values are determined under conditions when carbohydrates have not been ingested and are therefore independent of type of carbohydrate intake.

In the present study, reference δ^13^C data were obtained in each trial after 30 min of exercise before first intake of carbohydrates, thereby minimizing changes in isotopic composition of CO_2_ derived from endogenous fat oxidation, which progressively increases over time when no carbohydrates are ingested (32). Another methodological limitation when estimating exogenous carbohydrate oxidation rates from expired ^13^CO_2_ is the retention of ^13^CO_2_ in the circulating bicarbonate pool (57). Thus, it is possible that exogenous carbohydrate oxidation was underestimated during the 1st hour of exercise and that time for maximum oxidation, when identified, was overestimated in this present study.

## Conclusions

Ingestion of hypertonic MD+FRU drink, characterized by high fructose content and containing hydrogel-forming alginate and pectin, generated higher exogenous carbohydrate oxidation rates than isotonic MD+SUC (low fructose) and hypotonic AP (no fructose) drinks. Moreover, a less pronounced fall in dental biofilm pH with MD+FRU usage compared to that of MD+SUC and AP was evident, which may be favorable with respect to caries development. The long-term effects on dental health of frequent intake of commercially available sport drinks with differing acidulant content during both resting and active conditions warrants further research.

## Data Availability Statement

The raw data supporting the conclusions of this article will be made available by the authors on request.

## Ethics Statement

The studies involving human participants were reviewed and approved by the study received local ethics committee approval (Gothenburg University, Sweden) prior to any testing, and participants were informed about study details, both verbally and in writing, prior to providing written informed consent in accordance with the Declaration of Helsinki. The patients/participants provided their written informed consent to participate in this study.

## Author Contributions

SP, MA, and UA-H: conceptualization, data curation, and visualization. SP, FE, MA, PL, and CS: methodology. SP, FE, MA, and UA-H: formal analysis. SP and MA: writing–original draft preparation. SP, FE, MA, PL, CS, and UA-H: writing–review and editing. SP: project administration.

## Conflict of Interest

MA was employed by Maurten AB. The remaining authors declare that the research was conducted in the absence of any commercial or financial relationships that could be construed as a potential conflict of interest.
