# Corrigendum: A Hydrogel Drink With High Fructose Content Generates Higher Exogenous Carbohydrate Oxidation and a Reduced Drop in Dental Biofilm pH Compared to Two Other, Commercially Available, Carbohydrate Sports Drinks

**DOI:** 10.3389/fnut.2020.00128

**Published:** 2020-08-18

**Authors:** Stefan Pettersson, Martin Ahnoff, Fredrik Edin, Peter Lingström, Charlotte Simark Mattsson, Ulrika Andersson-Hall

**Affiliations:** ^1^Department of Food and Nutrition, and Sport Science, Center for Health and Performance, University of Gothenburg, Gothenburg, Sweden; ^2^Maurten AB, Research and Development, Gothenburg, Sweden; ^3^Department of Cariology, Institute of Odontology, Sahlgrenska Academy, University of Gothenburg, Gothenburg, Sweden; ^4^Department of Physiology, Institute of Neuroscience and Physiology, Sahlgrenska Academy, University of Gothenburg, Gothenburg, Sweden

**Keywords:** dental caries, endurance athletes, sports nutrition, stable isotope, substrate oxidation

In the original article, the title **“A Hydrogel Drink With High Fructose Content Generates Higher Exogenous Carbohydrate Oxidation and Lower Dental Biofilm pH Compared to Two Other, Commercially Available, Carbohydrate Sports Drinks”** was incorrectly written. It should be **“A Hydrogel Drink With High Fructose Content Generates Higher Exogenous Carbohydrate Oxidation and a Reduced Drop in Dental Biofilm pH Compared to Two Other, Commercially Available, Carbohydrate Sports Drinks”**.

In the original article, there was an error. The authors forgot to results on the Reproducibility of δ^13^CO_2_ measurements. The authors wish to add the second sentence below (in black) to the first paragraph in the Results, Exogenous and Endogenous Carbohydrate Oxidation section:

**“Exogenous carbohydrate oxidation (CHO_EXO_) for each participant (Supplementary Table 2) was calculated from changes in δ^13^CO_2_ of breath samples taken during the 3.5 h cycling exercise (Supplementary Figure 1). Reproducibility of δ^13^CO_2_ measurements was at an average 0.06‰ (range 0.03–0.09‰).”**

The authors apologize for this error and state that this does not change the scientific conclusions of the article in any way. The original article has been updated.

