# Supplementary material for: A Hydrogel Drink With High Fructose Content Generates Higher Exogenous Carbohydrate Oxidation and a Reduced Drop in Dental Biofilm pH Compared to Two Other, Commercially Available, Carbohydrate Sports Drinks
Source: Front Nutr. 2020 Jun 12;7:88. doi: 10.3389/fnut.2020.00088 (PMC7303329; doi:10.3389/fnut.2020.00088)
Supplement: Supplementary file 1 [file Data_Sheet_1.docx]

Supplementary Material

**Supplementary Table 1.**  δ ^13^CO_2_ (‰) in expired CO_2_ during 60 min fasted state exercise beginning with 30 min at 45% W_max_ and remaining time at 55% W_max_ (n=12).

|  | Time | |
| --- | --- | --- |
|  | 0-30 min | 30-60 min |
| Participant 1 | -27.41 | -27.59 |
| Participant 2 | -27.50 | -27.60 |
| Participant 3 | -27.32 | -27.02 |
| Participant 4 | -27.48 | -26.99 |
| Participant 5 | -28.07 | -28.06 |
| Participant 6 | -27.68 | -27.04 |
| Participant 7 | -26.64 | -26.84 |
| Participant 8 | -28.07 | -28.06 |
| Participant 9 | -27.88 | -27.87 |
| Participant 10 | -27.85 | -27.93 |
| Participant 11 | -27.66 | -27.58 |
| Participant 12 | -27.68 | -27.67 |
| **Mean** | **-27.60** | **-27.52** |
| SD | 0.39 | 0.44 |

**Supplementary Table 2.** Individual exogenous oxidation (g min^-1^) values during the 0‒180 min exercise (n=12) following repeated intake of maltodextrin+fructose (MD + FRU), maltodextrin+sucrose (MD + SUC) or amylopectin (AP) drinks.

| **MD + FRU** |  | | | | | | | | | | | |  |
| --- | --- | --- | --- | --- | --- | --- | --- | --- | --- | --- | --- | --- | --- |
| Time (min) | Participant 1 | Participant 2 | Participant 3 | Participant 4 | Participant 5 | Participant 6 | Participant 7 | Participant 8 | Participant 9 | Participant 10 | Participant 11 | Participant 12 | |
| 0 | 0 | 0 | 0 | 0 | 0 | 0 | 0 | 0 | 0 | 0 | 0 | 0 | |
| 20 | 0.245 | 0.098 | 0.186 | 0.150 | 0.066 | 0.050 | 0.261 | 0.377 | 0.205 | 0.100 | 0.108 | 0.162 | |
| 40 | 0.746 | 0.563 | 0.827 | 0.673 | 0.385 | 0.542 | 0.840 | 0.796 | 0.835 | 0.427 | 0.434 | 0.762 | |
| 60 | 1.036 | 0.943 | 1.139 | 1.212 | 1.023 | 0.752 | 1.142 | 1.029 | 0.974 | 0.812 | 0.831 | 0.958 | |
| 80 | 1.134 | 1.315 | 1.251 | 1.401 | 1.306 | 0.740 | 1.350 | 0.972 | 1.110 | 0.930 | 1.056 | 1.076 | |
| 100 | 1.294 | 1.389 | 1.333 | 1.514 | 1.375 | 0.861 | 1.403 | 1.314 | 1.317 | 1.011 | 1.024 | 1.130 | |
| 120 | 1.299 | 1.479 | 1.263 | 1.541 | 1.545 | 0.999 | 1.391 | 1.358 | 1.403 | 1.001 | 1.231 | 1.165 | |
| 140 | 1.276 | 1.405 | 1.260 | 1.453 | 1.493 | 0.965 | 1.347 | 1.237 | 1.429 | 1.209 | 1.350 | 1.159 | |
| 160 | 1.073 | 1.049 | 0.787 | 0.890 | 0.942 | 0.919 | 0.742 | 1.216 | 1.146 | 1.075 | 1.380 | 1.145 | |
| 180 | 0.737 | 0.639 | 0.361 | 0.570 | 0.437 | 0.929 | 0.494 | 0.705 | 0.668 | 0.898 | 1.019 | 0.764 | |

| **MD + SUC** |  | | | | | | | | | | | |  |
| --- | --- | --- | --- | --- | --- | --- | --- | --- | --- | --- | --- | --- | --- |
| Time (min) | Participant 1 | Participant 2 | Participant 3 | Participant 4 | Participant 5 | Participant 6 | Participant 7 | Participant 8 | Participant 9 | Participant 10 | Participant 11 | Participant 12 | |
| 0 | 0.000 | 0.000 | 0.000 | 0.000 | 0.000 | 0.000 | 0.000 | 0.000 | 0.000 | 0.000 | 0.000 | 0.000 | |
| 20 | 0.129 | 0.164 | 0.121 | 0.202 | 0.039 | 0.170 | 0.174 | 0.130 | 0.233 | 0.160 | 0.105 | 0.000 | |
| 40 | 0.440 | 0.499 | 0.440 | 0.846 | 0.251 | 0.816 | 0.630 | 0.471 | 0.553 | 0.551 | 0.476 | 0.608 | |
| 60 | 0.682 | 0.778 | 0.719 | 1.201 | 0.745 | 0.965 | 0.946 | 0.848 | 0.754 | 0.795 | 0.536 | 0.849 | |
| 80 | 0.883 | 0.969 | 0.945 | 1.297 | 1.021 | 1.091 | 1.110 | 1.025 | 0.907 | 0.927 | 0.794 | 0.903 | |
| 100 | 1.017 | 1.052 | 1.082 | 1.338 | 1.063 | 1.203 | 1.234 | 1.035 | 0.956 | 0.947 | 1.006 | 0.983 | |
| 120 | 1.103 | 1.162 | 1.168 | 1.328 | 1.113 | 1.269 | 1.409 | 1.061 | 1.079 | 0.988 | 1.113 | 0.994 | |
| 140 | 1.273 | 1.162 | 1.126 | 1.434 | 1.179 | 1.255 | 1.445 | 1.079 | 1.055 | 1.045 | 1.128 | 0.976 | |
| 160 | 1.278 | 1.265 | 1.091 | 1.285 | 1.248 | 1.241 | 1.354 | 1.119 | 1.056 | 1.031 | 1.214 | 1.085 | |
| 180 | 1.246 | 1.214 | 1.142 | 0.630 | 1.266 | 1.278 | 0.594 | 1.083 | 1.100 | 1.084 | 1.119 | 0.973 | |

| **AP** |  | | | | | | | | | | | |  |
| --- | --- | --- | --- | --- | --- | --- | --- | --- | --- | --- | --- | --- | --- |
| Time (min) | Participant 1 | Participant 2 | Participant 3 | Participant 4 | Participant 5 | Participant 6 | Participant 7 | Participant 8 | Participant 9 | Participant 10 | Participant 11 | Participant 12 | |
| 0 | 0.000 | 0.000 | - | 0.000 | 0.000 | 0.000 | 0.000 | 0.000 | 0.000 | 0.000 | 0.000 | 0.000 | |
| 20 | 0.126 | 0.119 | - | 0.140 | 0.080 | 0.181 | 0.158 | 0.186 | 0.247 | 0.103 | 0.184 | 0.078 | |
| 40 | 0.505 | 0.294 | - | 0.548 | 0.123 | 0.483 | 0.464 | 0.476 | 0.534 | 0.312 | 0.616 | 0.473 | |
| 60 | 0.682 | 0.678 | - | 0.958 | 0.483 | 0.771 | 0.692 | 0.720 | 0.672 | 0.650 | 0.783 | 0.730 | |
| 80 | 0.844 | 0.902 | - | 0.986 | 0.593 | 0.967 | 0.850 | 0.829 | 0.774 | 0.824 | 0.861 | 0.719 | |
| 100 | 0.868 | 0.910 | - | 1.063 | 0.697 | 1.042 | 0.986 | 0.852 | 0.865 | 0.838 | 0.895 | 0.765 | |
| 120 | 0.922 | 1.051 | - | 1.171 | 0.734 | 1.057 | 1.038 | 0.875 | 0.901 | 0.879 | 0.935 | 0.830 | |
| 140 | 0.895 | 1.160 | - | 1.163 | 0.884 | 1.005 | 1.104 | 0.925 | 0.843 | 0.892 | 1.004 | 0.890 | |
| 160 | 0.919 | 1.183 | - | 1.192 | 0.921 | 1.143 | 1.048 | 0.942 | 0.873 | 0.952 | 0.931 | 0.874 | |
| 180 | 0.850 | 1.211 | - | 1.188 | 1.001 | 1.143 | 1.104 | 0.898 | 0.971 | 0.948 | 0.916 | 0.897 | |


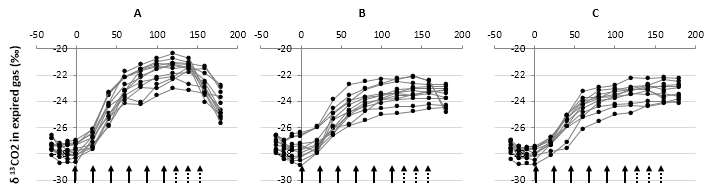


**Supplementary Figure 1.** Spaghetti plot of individual changes (n=12) in δ ^13^CO_2_ (‰) in expired CO_2_ following repeated ingestion of (A) MD+FRU, (B) MD+SUC, and (C) AP during the 3.5 h of cycling, where MD+FRU is a maltodextrin+fructose drink, MD+SUC maltodextrin+sucrose drink and AP amylopectin drink.
Note: carbohydrate intake, filled arrows; and plain water intake, dashed arrows.

**Supplementary Text**. Estimation of error in calculation of exogenous oxidation for the maltodextrin + sucrose (MD+SUC) drink

Measuring exogenous carbohydrate oxidation relies on the isotopic enrichment of δ ^13^C in the ingested carbohydrates compared to endogenous energy sources. For a carbohydrate mixture, the measured δ ^13^C is used for calculation. When the enrichment is closely similar for different carbohydrates ingested simultaneously, the calculated exogenous carbohydrate oxidation will correctly reflect the total oxidation, while the contribution (oxidation efficiency) of each carbohydrate will not necessarily be the same.

If the isotopic enrichment for one carbohydrate ingredient is lower than that of the other(s), the calculated oxidation will correctly reflect the total oxidation of the carbohydrates, as long as the oxidation efficiency is the same for all of the carbohydrates. In this study, fructose and maltodextrin were both derived from corn and so had similar isotope enrichment. The isotopic enrichment of sucrose used in the MD+SUC drink was lower, as evident from the measured δ ^13^C (-14.67 o/oo) that was in accord with sucrose not being derived from sugar cane, but rather from sugar beet. Since the isotopic enrichment for beet sugar was virtually the same as the background enrichment (endogenous sources), the estimation of total exogenous oxidation will be erroneous if the oxidation efficiency for sucrose differed from that of co-ingested maltodextrin.

It can be assumed that the sucrose (20% of total carbohydrates) was rapidly metabolized to equal amounts of glucose and fructose. Assuming that fructose had a 10% higher oxidation efficiency than maltodextrin (85% instead of 75%, see also Figure 2), underestimation of the contribution of sucrose to the total oxidation would be in the order of 1%, as fructose amounted to one tenth of the total carbohydrates.
